# Network pharmacology and experimental validation to identify the potential mechanism of *Hedyotis diffusa* Willd against rheumatoid arthritis

**DOI:** 10.1038/s41598-022-25579-3

**Published:** 2023-01-25

**Authors:** Hui Deng, Jing Jiang, Sisi Zhang, Lijuan Wu, Qinglian Zhang, Wenkui Sun

**Affiliations:** 1 School of Laboratory Medicine, Chengdu Medical College, Chengdu, 610500 Sichuan China; 2 Department of Library, Chengdu Medical College, Chengdu, 610500 Sichuan China

**Keywords:** Biochemistry, Health occupations, Rheumatology

## Abstract

Rheumatoid arthritis (RA) is a chronic, systemic, autoimmune disease that may lead to joint damage, deformity, and disability, if not treated effectively. *Hedyotis diffusa* Willd (HDW) and its main components have been widely used to treat a variety of tumors and inflammatory diseases. The present study utilized a network pharmacology approach, microarray data analysis and molecular docking to predict the key active ingredients and mechanisms of HDW against RA. Eleven active ingredients in HDW and 180 potential anti-RA targets were identified. The ingredients-targets-RA network showed that stigmasterol, beta-sitosterol, quercetin, kaempferol, and 2-methoxy-3-methyl-9,10-anthraquinone were key components for RA treatment. KEGG pathway results revealed that the 180 potential targets were inflammatory-related pathways with predominant enrichment of the AGE-RAGE, TNF, IL17, and PI3K-Akt signaling pathways. Screened through the PPI network and with Cytoscape software, RELA, TNF, IL6, TP53, MAPK1, AKT1, IL10, and ESR1 were identified as the hub targets in the HDW for RA treatment. Molecular docking was used to identify the binding of 5 key components and the 8 related-RA hub targets. Moreover, the results of network pharmacology were verified by vitro experiments. HDW inhibits cell proliferation in MH7A cells in a dose and time-dependent manner. RT-qPCR and WB results suggest that HDW may affect hub targets through PI3K/AKT signaling pathway, thereby exerting anti-RA effect. This study provides evidence for a clinical effect of HDW on RA and a research basis for further investigation into the active ingredients and mechanisms of HDW against RA.

## Introduction

Rheumatoid arthritis (RA), one of the most common autoimmune diseases, is characterized by invasive joint synovitis, pannus formation, deterioration of joints, and loss of joint function^1,2^. RA occurs in 0.5 to 1.0% of the population worldwide^3^. In China, up to 5 million people suffer from pain and recurrence of RA^4^. Due to its high prevalence, debilitating nature, and disabling consequences, RA has generated a substantial clinical, economic, and social burden. The pathogenesis of RA is complicated, incompletely understood, and considered to be mediated by various mechanisms. At present, the typical events in the pathogenesis of RA are considered to be hyperplasia of cells in the synovial membrane consisting of synovial fibroblasts, macrophages, and lymphocytes^5^. Some studies have shown that fibroblast-like synoviocytes (FLS) are the dominant cell type and are considered to play a critical role in the pathogenesis of RA^6,7^. Disease-modifying antirheumatic drugs (DMARDs) and non-steroidal anti-inflammatory medications (NSAIDs) have been widely used in RA therapy^8^. Conventional DMARDs, including hydroxychloroquine, methotrexate (MTX), sulfasalazine, and leflunomide, have been approved by the US Food and Drug Administration (FDA) as a first-line therapy for RA patients^9^; however, there are no truly effective pharmacotherapies for the treatment of RA. Most of these drugs have frequent side effects, including gastrointestinal irritation, kidney injury, and cardiovascular risk^10^. There is therefore an urgent need for safe and effective medical treatments for RA.

In recent years, with the understanding of pathogenesis of RA in Chinese medicine, some progress has been made in the treatment of RA with traditional Chinese medicine. *Hedyotis Diffusa* Willd (HDW) is a member of the Rubiaceae family of Chinese herbal remedies and is mainly found in the southeastern provinces of China^11^. Modern pharmacological studies have shown that HDW exhibits multiple pharmacological effects, including anti-tumor, anti-inflammatory, anti-oxidation, anti-fibroblastic, hepatoprotective and immunomodulatory^12,13^. It has been widely studied as a potential therapeutic drug for treatment of malignant tumors of the breast, stomach, colon, rectum, cervix, and ovary^14–16^. It has also been used in the treatment of inflammation-related diseases, including urinary tract infection, colitis, tonsillitis, appendicitis, pharyngitis, hepatitis, dysentery, diarrhea, and snake bites^17,18^. Studies have shown that the certain chemical constituents of HDW (scandoside, asperuloside and asperulosidic acid) exerted an anti-inflammatory effect on LPS-induced RAW 264.7 macrophages by suppressing the NF-κB and MAPK signaling pathways^19,20^. In a complete Freund's adjuvant (CFA)-induced arthritis model in rats, 12 days of oral treatment with HDW extract ursolic acid (50 mg/kg/day) was demonstrated to suppress paw swelling, plasma PGE (2) production, spinal Fos expression, and arthritis-induced mechanical and thermal hyperalgesia^21^. Zhu et al.^22,23^ also revealed that HDW compounds ferulic acid and p-coumaric acid demonstrated an anti-inflammatory effect on collagen-induced arthritis as indicated by decreased numbers of inflammatory cells and reduced levels of IL-1β and TNF-α. Intriguingly, unfractionated HDW had a better therapeutic outcome than ferulic acid, although a poorer one than p-coumaric acid alone. A recent study has shown that HDW effectively suppressed the progression of disease in a collagen-induced arthritis (CIA) model by reducing the arthritis index, by reducing levels of IL-lβ, TNF-α, PGE2, RANKL, OPG, and RANKL/OPG, and by increasing the pain threshold^24^. Nevertheless, despite extensive studies on the pharmacological effect of HDW, the potential targets and underlying molecular mechanism(s) of HDW in RA remain unclear.

**Figure 1:**
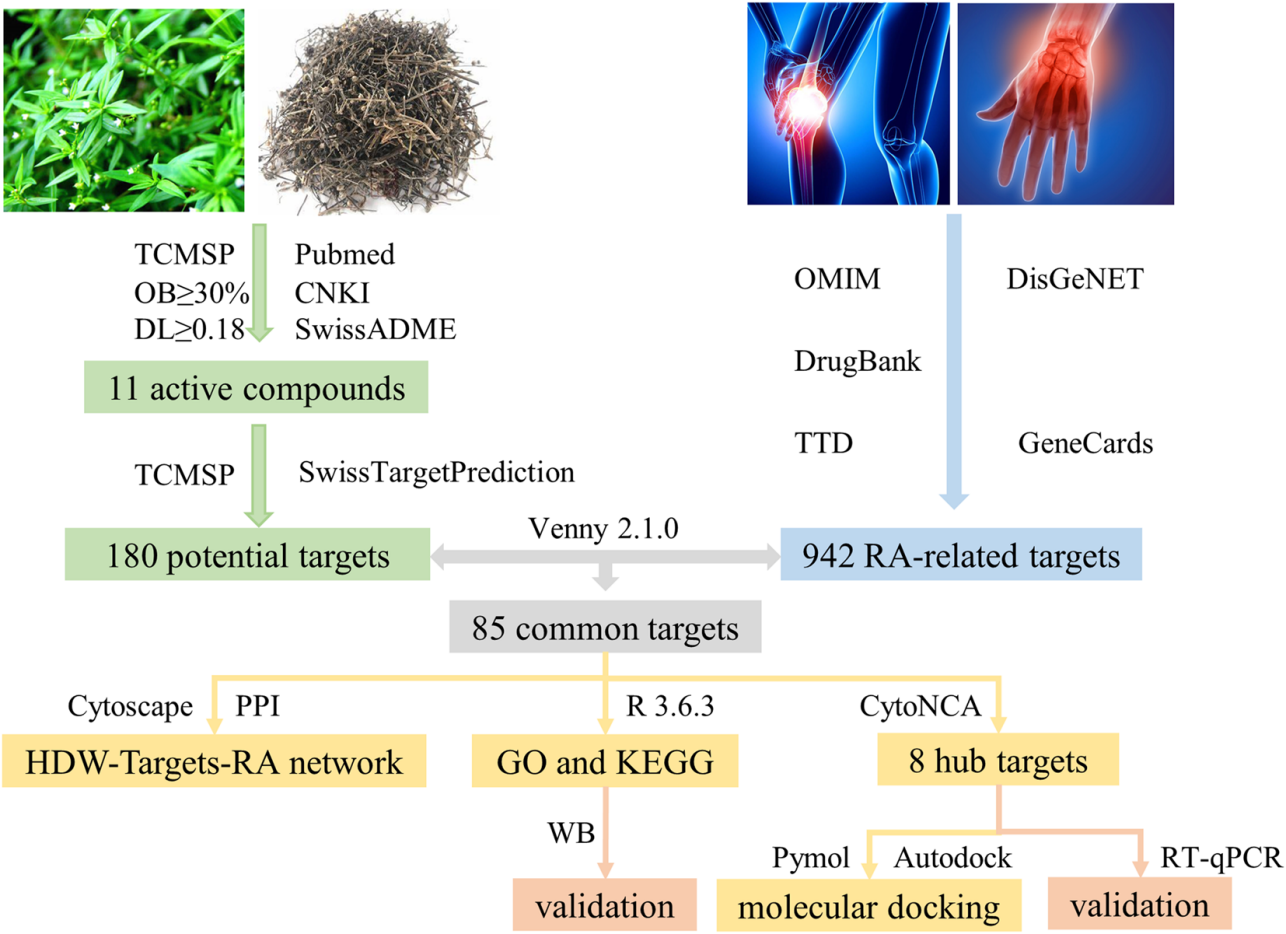
A flow-chart of this study to investigate the potential mechanism of HDW in treatment of RA.

Network pharmacology is a novel approach that combines system network analysis and pharmacology^25^. Through network pharmacological analyses, we can investigate TCM systematically, identify the active components, predict potential targets and mechanisms, and provide the opportunity for modernization of TCM^26^. Therefore, our study aimed to investigate the active ingredients, potential targets, and the underlying mechanism of HDW for the treatment of RA by adopting a network pharmacology approach, molecular docking and cell experiments. The main scheme of this study is presented in Fig. 1. We screened public databases (TCMSP) and published literature to identify the active ingredients of HDW. Then, network pharmacology was used to analyze ingredients targets, drug targets, biological processes and pathways in RA treatment. In addition, we also used cell experiments to identify the results of network pharmacology.

## Results

### Active components and potential targets of HDW

Our study performed network pharmacology prediction based on network pharmacology evaluation method guidance‐Draft^27^. A total of 142 related components of HDW was retrieved from TCMSP and the published literature. According to pharmacokinetic characteristics (OB ≥ 30% and DL ≥ 0.18) and ADME information, 11 active components were selected from 142 ingredients of HDW. The TCMSP and Swiss Target Prediction databases were used to determine the pharmacological targets of the HDW components. Table 1 shows active components and the number of the corresponding potential targets of HDW. Detailed information of these components and targets is listed in Supplementary Table 1. Eventually, 180 potential targets were identified (after removing duplicates) using the Uniprot database.

**Table 1:** Active components and numbers of corresponding potential HDW targets.

PubChem CID	Active components	Target number
5281330	Poriferasterol	2
10514946	2-Methoxy-3-methyl-9,10-anthraquinone	31
5280794	Stigmasterol	31
222284	β-Sitosterol	38
5280343	Quercetin	154
5280863	Kaempferol	17
5280460	Scopoletin	3
637542	p-Coumaric acid	13
72	3, 4-Dihydroxybenzoic acid	8
445858	Ferulic acid	8
135	p-Hydroxybenzoic acid	12

### Identification of the potential targets of RA

Using “Rheumatoid Arthritis” as the search term, 42, 192, 141, 174, and 623 disease-targets were obtained from the OMIM, DrugBank, TTD, GeneCards, and DisGeNET databases, respectively. Merging all results from the five databases and removing duplicates, 942 related-RA potential targets were finally collected.

### Construction of the active components-common targets-RA network

**Figure 2:**
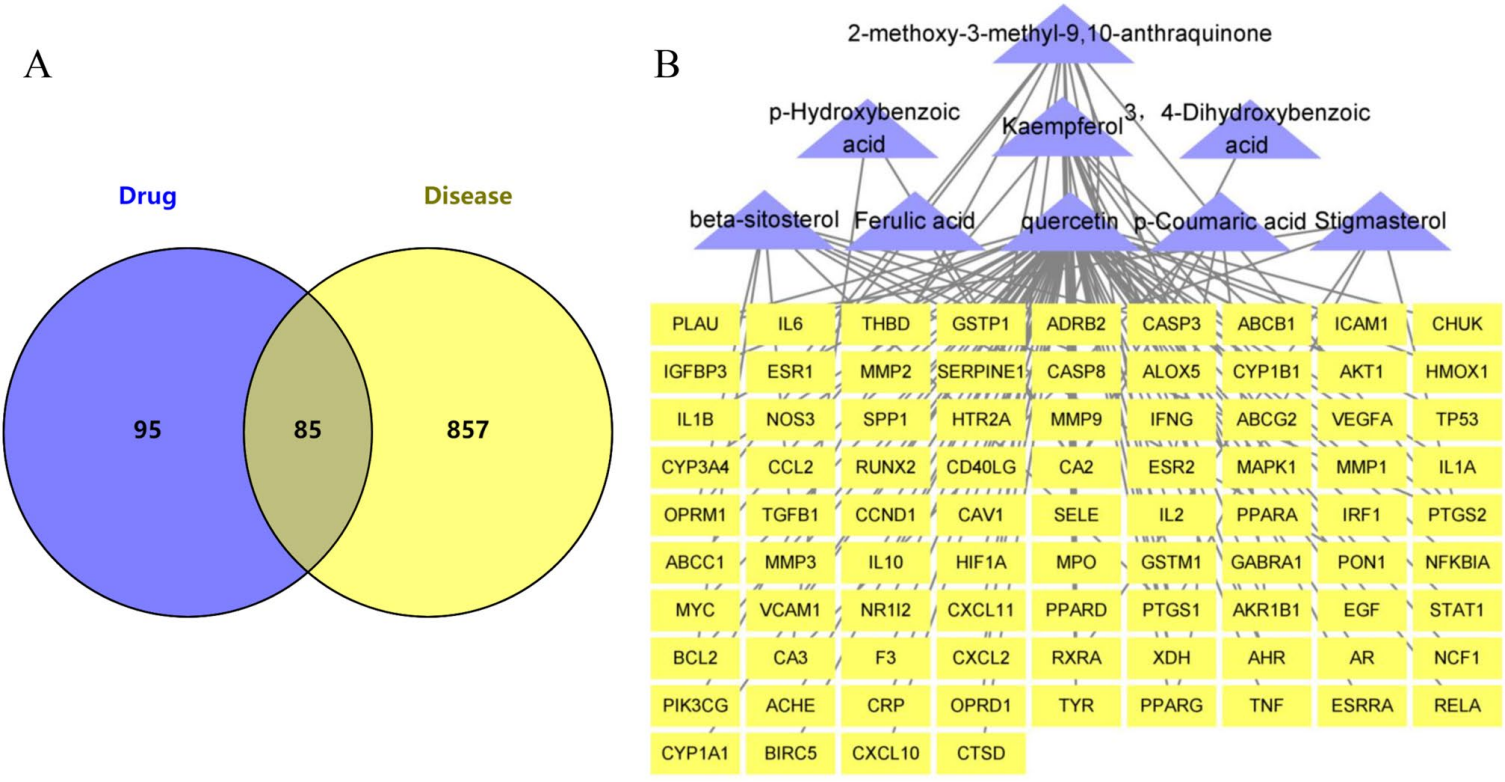
Construction of the components-targets-RA Network. (**A**) Venn diagram of active ingredients of HDW and RA targets. (**B**) Active ingredients-targets-RA network. Purple triangles represent 9 components of HDW and yellow rectangles represent common targets.

Applying a Venn diagram, 85 common targets were found overlapped between HDW compound targets and RA-related targets (Fig. 2A). We imported 11 active components and 85 common targets into Cytoscape 3.9.0 software to construct a components-targets-RA network. Among these, the active ingredients with the highest degree value were stigmasterol, β-sitosterol, quercetin, kaempferol and 2-methoxy-3-methyl-9,10-anthraquinone. However, poriferasterol and scopoletin were removed since they lacked common targets in the network. The active ingredients-targets-RA network is shown in Fig. 2B. Results indicate that 5 components may provide the key to successful treatment of RA.

### GO and KEGG enrichment analysis

**Figure 3:**
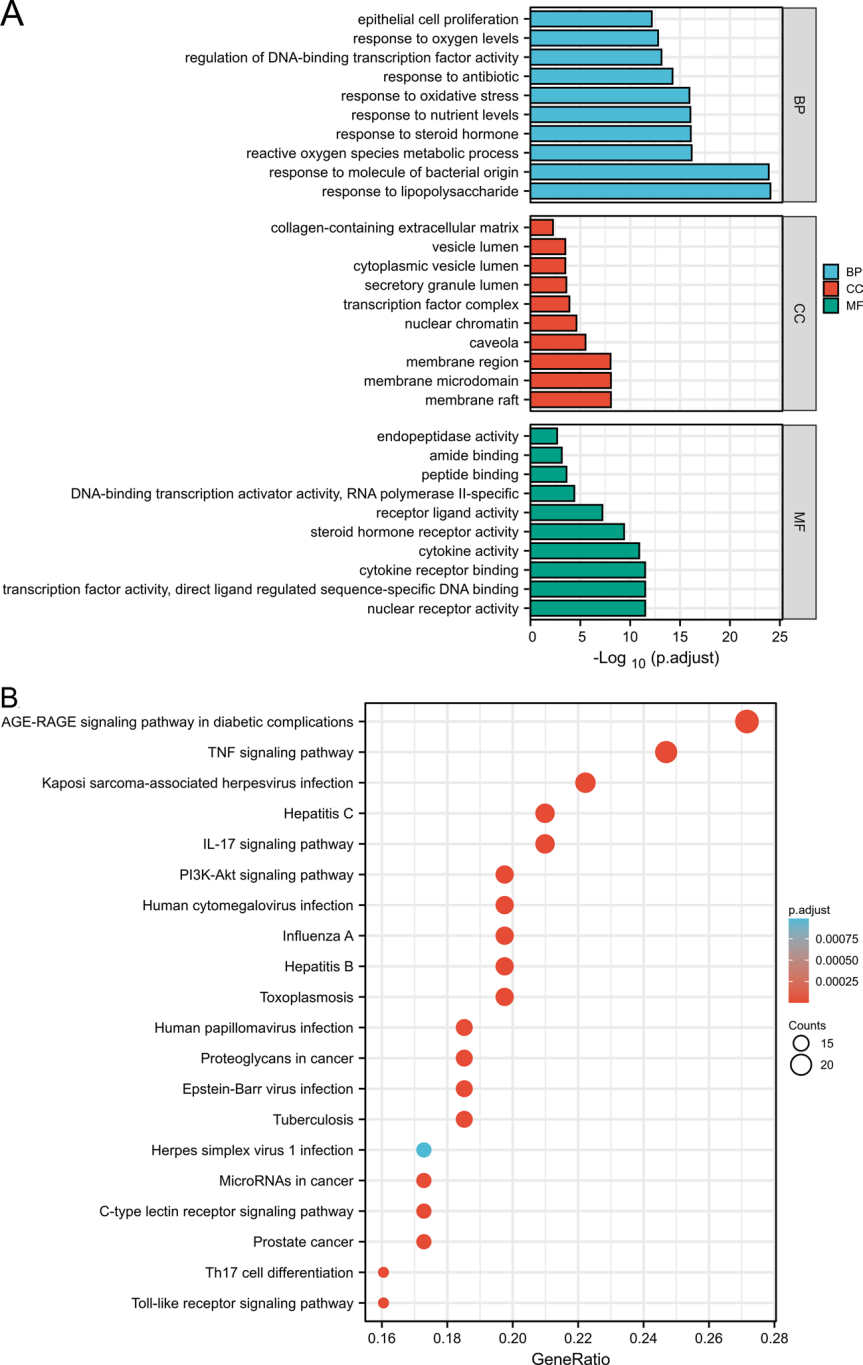
GO and KEGG analysis of potential targets. (**A**) Different colors represent different categories. The height of the column represents the P.adjust value: the higher the value, the higher the reliability of the GO categories (P.adjust < 0.01). (**B**) Dot size indicates the number of target genes in the pathway, and dot color reflects the different P.adjsted value ranges.

In total, GO analysis identified 1542 significantly enriched GO terms (P.adjusted < 0.01 adjusted with Benjamini–Hochberg), consisting mainly of 1459 biological processes, 18 cellular components, and 65 molecular functions. We screened the top 10 ranked GO terms shown in Fig. 3A. In the biological process (GO:BP) category, the top terms were involved in responses to lipopolysaccharides, molecules of bacterial origin, and reactive oxygen species metabolic processes. In the cellular component (GO:CC) category, the top terms included membrane rafts, membrane microdomains, and membrane regions. In the molecular function (GO:MF) category, the top terms consisted of nuclear receptor activity, transcription factor activity, and cytokine receptor binding. To further identify underlying signaling pathways, we analyzed KEGG pathways. The top 20 significantly enriched pathways (P.adjusted value < 0.01) are shown in Fig. 3B. A list of genes contributing to the 20 selected pathways is provided in Supplementary Table 2. Numerous targets were found associated with the AGE-RAGE, TNF, IL17, and PI3K-Akt signaling pathways, all of which are associated with the prognosis and onset of RA.

### Network visualization and identification of hub targets

**Figure 4:**
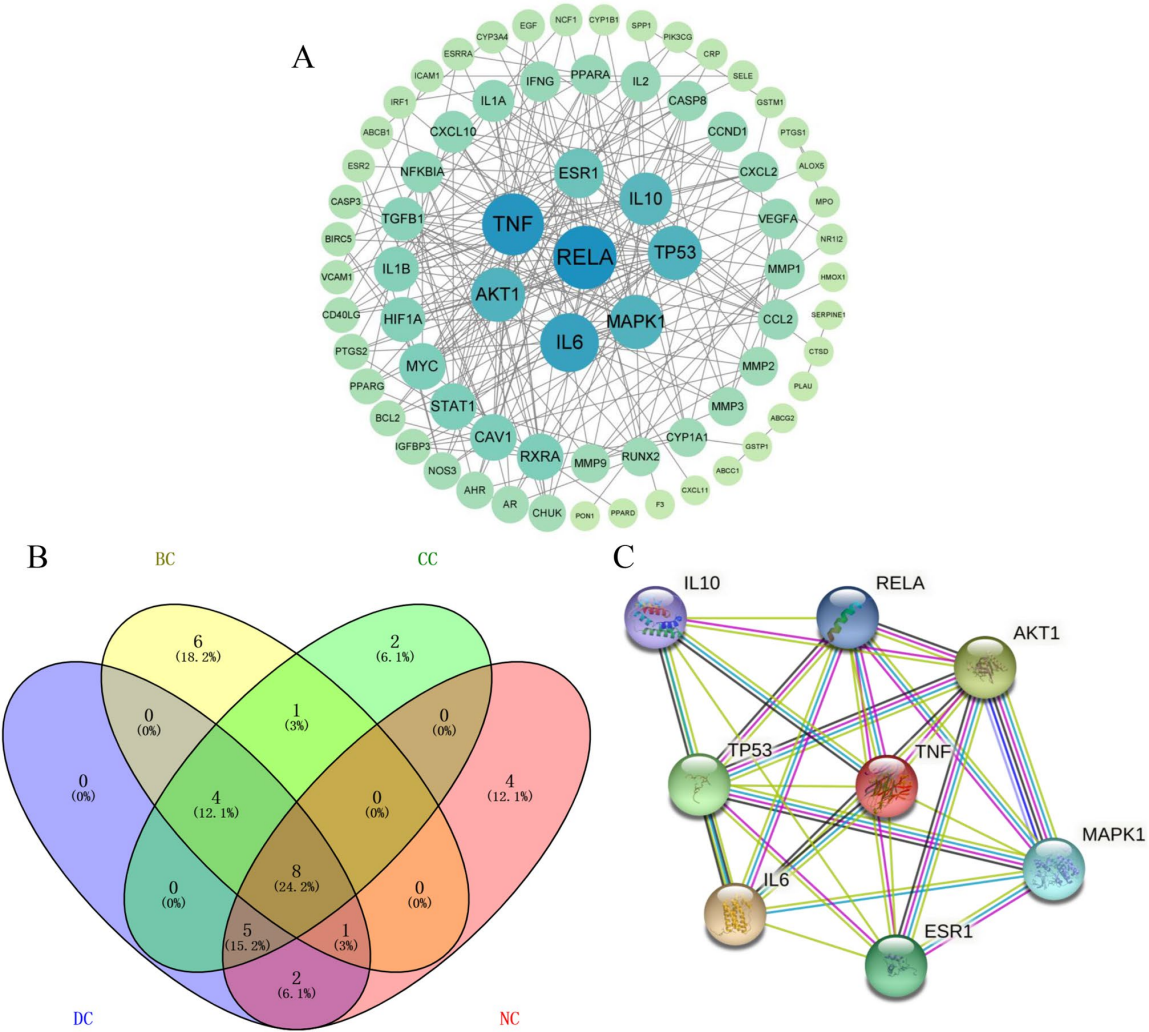
Construction of the PPI network and screening hub targets. (**A**) PPI network of 85 common targets. (**B**) Hub genes were screened from the PPI network using the Betweenness (BC), Closeness (CC), Degree (DC), and Network (NC) methods. (**C**) Subnetwork of the PPI network of 8 hub targets. The color and size of the nodes reflect the degree value for each protein target: the larger and darker the node, the greater the degree value. The different colored lines in the figure represent known interactions and predicted interactions (light blue: from curated databases; dark purple: experimentally determined; green: gene neighborhood; red: gene fusions; dark blue: gene co-occurrence; yellow: textmining; black: co-expression; light purple: protein homology).

Next, we analyzed 85 potential therapeutic targets by using the STRING database to obtain a PPI network to explore the relationship between RA-related targets. The PPI relationship network, with a total of 85 nodes, 238 edges and an average node degree of 5.6 was generated with a confidence of 0.9 (Supplementary Fig. 1). PPI network diagrams were imported into Cytoscape 3.9.0 software for visualization (Fig. 4A). We further identified the subnetwork and hub targets from the PPI network using the CytoNCA plug-in (Fig. 4B). As shown in Fig. 4C, a subnetwork was identified, including 8 nodes and 27 edges. Moreover, RELA, TNF, IL6, TP53, MAPK1, AKT1, IL10, and ESR1 were identified as the hub targets in the HDW for RA treatment (Supplementary Table 3).

### Molecular docking

**Figure 5:**
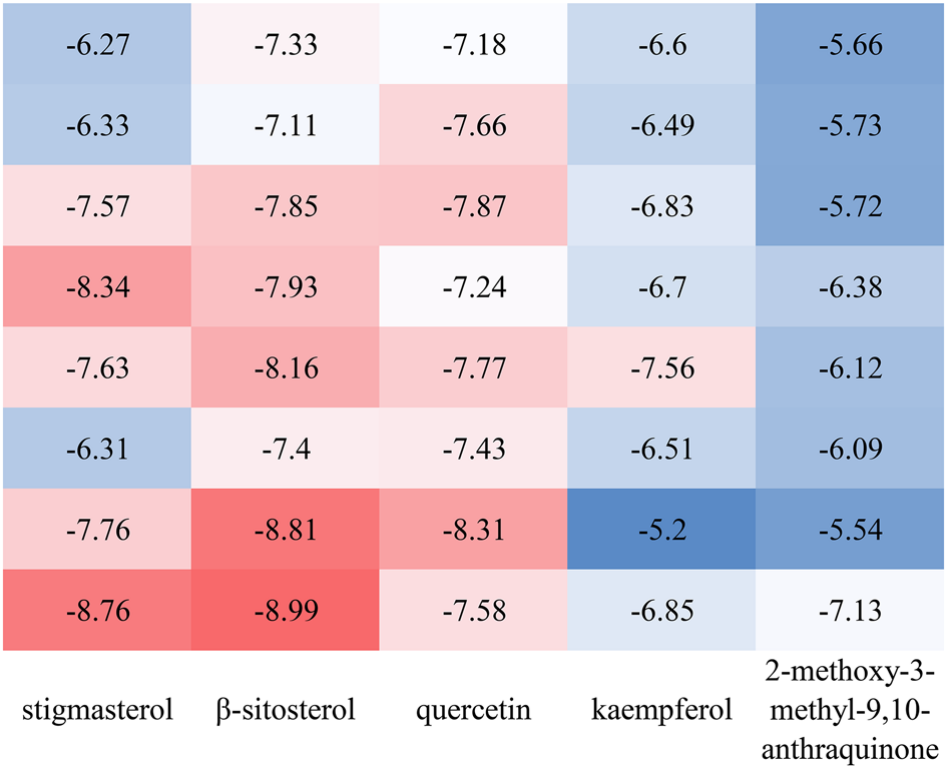
Molecular docking heatmap of the main compounds and key targets (kcal/mol). The figure shows the size of the binding energy. The larger the absolute value, the redder the color, indicating increasing stability of the combination of the component and the target protein.

**Figure 6:**
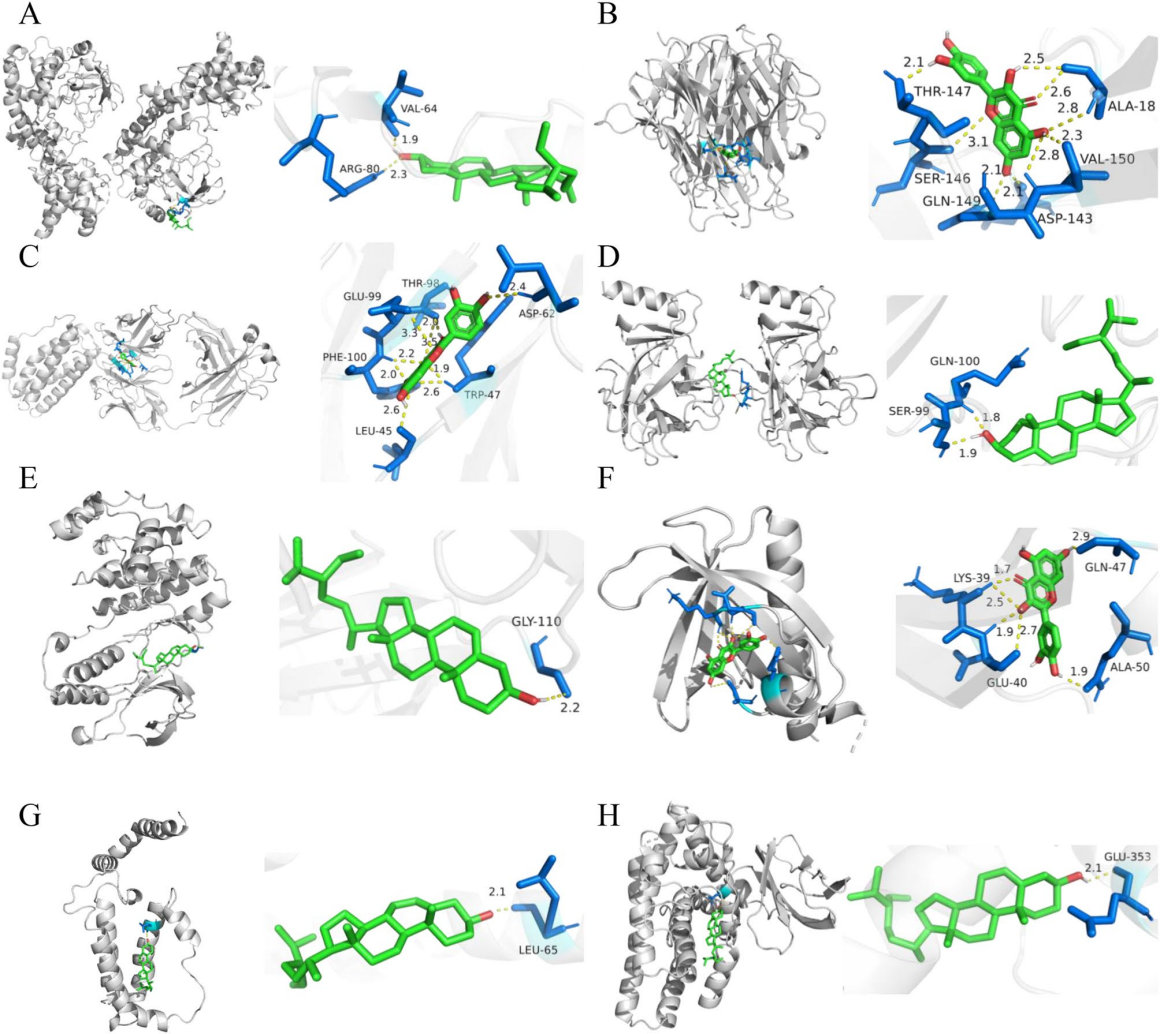
Docking complexes of ligand and receptor proteins and their binding residues are shown using PYMOL software. (**A**) RELA and β-sitosterol. (**B**) TNF and quercetin. (**C**) IL6 and quercetin. (**D**) TP53 and stigmasterol. (**E**) MAPK1 and β-sitosterol. (**F**) AKT1 and quercetin. (**G**) IL10 and β-sitosterol. (**H**) ESR1 and β-sitosterol.

Candidate compounds stigmasterol, β-sitosterol, quercetin and kaempferol, and 2-methoxy-3-methyl-9,10-anthraquinone, are the top 5 (ranked by degree) in the compounds-targets-RA network. The hub targets, RELA, TNF, IL6, TP53, MAPK1, AKT1, IL10, and ESR1, play a significant role in the action of HDW against RA. Molecular docking of the 5 compounds and 8 hub genes revealed binding energies shown in Fig. 5. Five components of HDW exhibited strong binding to the 8 core targets with β-sitosterol showing the highest binding energy. these results imply that treatment with HDW may affect all the Figure targets in RA patients. The target proteins and the small molecules with strong binding affinity were visualized by PyMoL software (Fig. 6).

### Cell experiments

**Figure 7:**
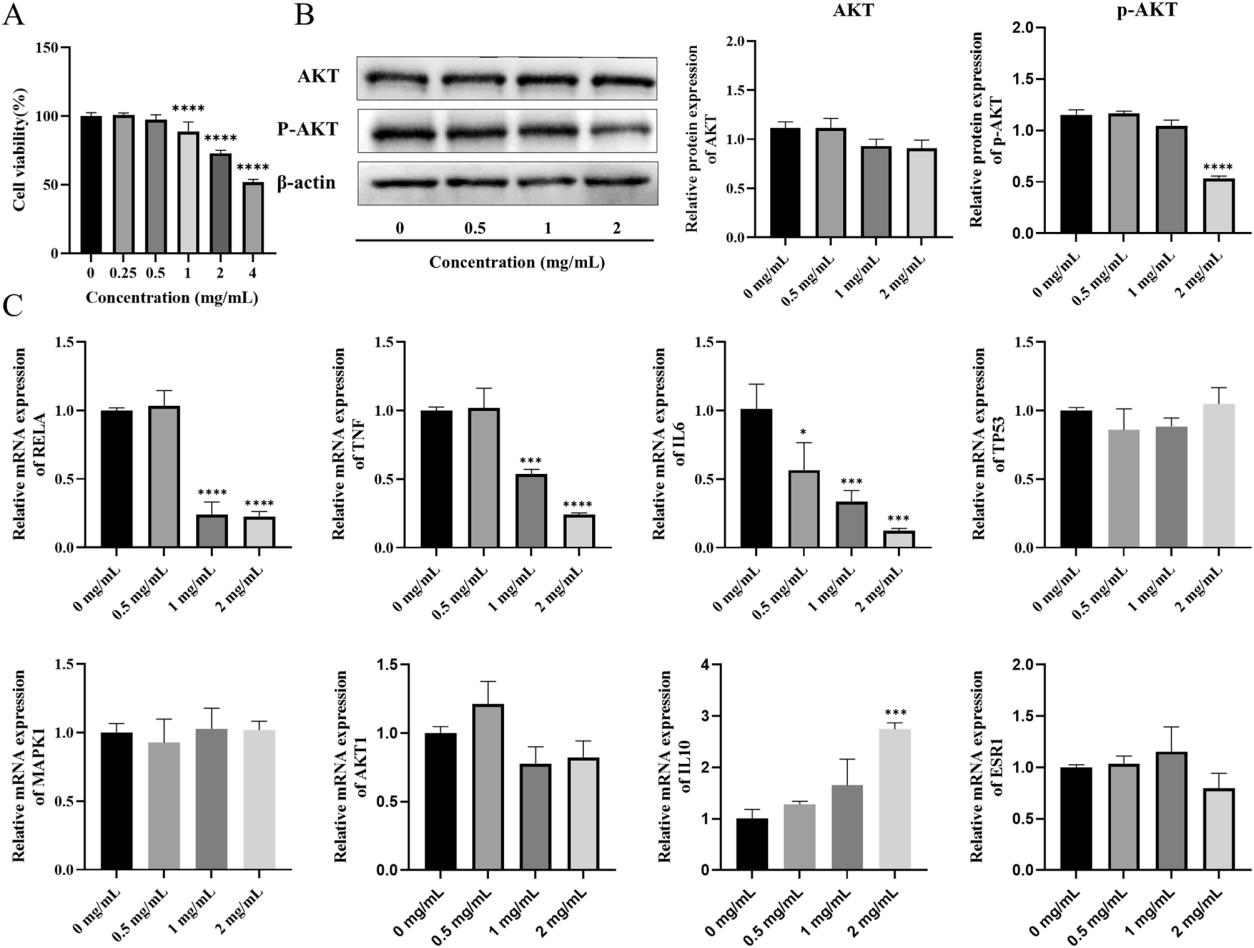
Cell experiments validate results of network pharmacology. (**A**) CCK8 assays of different HDW concentrations (0, 0.5, 1, 2 mg/mL) incubated MH7A cells for 48 h. (**B**) The expression levels of AKT and p-AKT were measured using western blotting. Original blots are presented in Supplementary Fig. 2. (**C**) The effect of HDW on the mRNA levels of RELA in MH7A cells. Compared with control (0 mg/mL), *P < 0.05, **P < 0.01, ***P < 0.001, ****P < 0.0001.

Overproliferation of fibroblast-like synoviocytes (FLS) are important pathogenesis of RA. Therefore, we investigated the effect of HDW at different concentrations (0, 0.5, 1, 2 mg/mL) on the proliferation of MH7A cells after 48 h. CCK-8 assay showed that HDW inhibited the proliferation of MH7A cells in a dose-dependent (Fig. 7A). According to the results, we chose to perform subsequent experiments with the dose of 0.5, 1, and 2 mg/mL. The WB result showed that HDW could inhibit the PI3K/AKT pathway by reducing phosphorylation of AKT in MH7A cells (*P* < 0.05, Fig. 7B). Hub targets in our work were verified by RT-qPCR and results showed that RELA, TNF, and IL6 were up-regulated while IL10 was down-regulated (Fig. 7C). These results validated our network pharmacology analysis, suggesting that HDW can play a role in treating RA by regulating PI3K/AKT signaling pathway and RA-related targets.

## Discussion

Given their potent anti-inflammatory, anti-fibroblastic, and immunomodulatory actions, HDW and its components have been widely used in animal models over the last several years as an intervention for RA^12,13,19–23^. Unfortunately, the potential targets and molecular mechanism of HDW against RA are still inadequately understood. TCM network pharmacology emerging recently has become a flourishing field in TCM modern studies along with the rapid progress of bioinformatics. The study of TCM and biological network appeared for the first time in 2007^28^, and some applications of traditional medicine network pharmacology for herbs or herbal formulae in RA^29^. In the present network pharmacological analysis, a total of 142 compounds of HDW were identified from TCMSP and published literature, and 11 compounds were selected by TCMSP and ADME criteria screening. A total of 180 targets related to potential compounds and 942 targets associated with RA were identified, and 85 common target genes were obtained from the overlapping part of identified compounds and RA. The components-targets-RA network analysis visualized the interaction of multi-components and multitargets about HDW on RA. The compounds targets network analysis indicated that the 5 compounds, including stigmasterol, β-sitosterol, quercetin, kaempferol, and 2-methoxy-3-methyl-9,10-anthraquinone, were linked to ≥ 10 target genes, and the 8 target genes (RELA, TNF, IL6, TP53, MAPK1, AKT1, IL10, and ESR1) were core target genes in the network. GO enrichment analysis indicated that numerous targets are involved in response to lipopolysaccharides and molecules of bacterial origin in BP, are localized to membrane rafts and membrane microdomains in CC, and are associated with nuclear receptor and transcription factor activities in MF. KEGG pathway analysis indicated that numerous targets are associated with certain inflammatory events and cancer. Molecular docking showed that stigmasterol, β-sitosterol, quercetin, kaempferol, and 2-methoxy-3-methyl-9,10-anthraquinone have good binding activity with RELA, TNF, IL6, TP53, MAPK1, AKT1, IL10, and ESR1 targets. Finally, the molecular mechanisms of HDW predicted by network pharmacology approach against RA were validated by in vitro experiments.

A potential components-targets-RA target network indicated that stigmasterol, β-sitosterol, quercetin, kaempferol, and 2-methoxy-3-methyl-9,10-anthraquinone, are likely to play vital roles in the process of RA treatment (Table 2). Indeed, apart from 2-methoxy-3-methyl-9,10-anthraquinone, all these components have previously been reported to exhibit potential antirheumatic therapeutic activity. For example, stigmasterol has been shown to protect CIA rats by suppressing proinflammatory mediators (TNF-α, IL-6, IL-1β, iNOS and COX-2) and increasing anti-inflammatory cytokine IL-10^30^. β-sitosterol exerts an inhibitory influence on synovial angiogenesis by suppressing endothelial cell proliferation and migration, thereby alleviating joint swelling and bone destruction in CIA mice^31^, and quercetin inhibits the release of proinflammatory cytokines (IL-6, TNF-α, IL-1β, IL-8, IL-13, IL-17) by activating SIRT1, thereby becoming a potential effector of RA^32,33^. Kaempferol inhibits the proliferation and migration of RA-FLSs and the release of activated T-cell-mediated inflammatory cytokines by suppressing fibroblast growth factor receptor 3-ribosomal S6 kinase 2 (FGFR3-RSK2) signaling^34^. Collectively, these active components exhibit antirheumatic activity by various mechanisms, including anti-inflammatory, immunoregulatory, and reduction of bone destruction. Notably, however, there have been few previous studies on the treatment of RA with stigmasterol and β-sitosterol.

**Table 2:** Potential anti-RA mechanisms of some compounds.

Compound	Mechanism	Model	References
stigmasterol	Suppressed proinflammatory mediators and increased anti-inflammatory cytokine through down-regulating NF-kB, p65 and p38MAPK	CIA	Ahmad et al. (2020)^30^
β-Sitosterol	Inhibited synovial angiogenesis by suppressing endothelial cells proliferation and migration	CIA	Qian et al. (2022)^31^
	Repressed the M1 polarization and augmented M2 polarization	mø	Liu et al. (2019)^48^
Quercetin	Inhibited the release of proinflammatory cytokine (IL-6, TNF-α, IL-1β, IL-8, IL-13, IL-17) by activating SIRT1	CIA	Shen et al. (2021)^49^
	Inhibited NLRP3 inflammasome activation and activated HO-1-mediated anti-inflammatory response via modulating the Th17/Treg balance	CIA	Yang et al. (2017)^50^
	Inhibited IL-17 and RANKL production, suppressed Th17 cell	FLS	Kim et al. (2019)^33^
	Modulated the immune response to arthritis via attenuation of the purinergic system (E-NTPDase and E-ADA activities) and the levels of IFN-γ and IL-4	CFA	Saccol et al. (2019)^51^
Kaempferol	Inhibited RAFLS proliferation, migration, and inflammatory cytokines by suppressing FGFR3-RSK2 signaling	FLS	Lee et al. (2018)^34^
	Inhibited RAFLS migration and invasion by blocking MAPK pathway	FLS	Pan et al. (2017)^52^
	Enhanced the suppressive function of Treg cells by inhibiting FOXP3 phosphorylation	Treg	Lin et al. (2015)^53^
	Inhibited RAFLS proliferation, reduced MMPs, COX-2, and PGE2 production, inhibited NF-κB activation	FLS	Yoon et al. (2013)^54^

KEGG pathway analysis indicated that the 85 therapeutic targets were enriched in viral infection and cancer, such as Kaposi’s sarcoma-associated herpesvirus infection, human cytomegalovirus infection, human papillomavirus infection, and prostate cancer. The evidence that viral infection such as human cytomegalovirus infection^35^ contributes to RA is strong, and that RA is associated with an increased risk of cancer^36^. Additionally, the KEGG analysis results also indicated that the AGE-RAGE, TNF, IL-17, and PI3K-Akt signaling pathways may be critical in the network pharmacology. It has been shown that the PI3K/AKT signaling pathway, the key molecular mechanism of the occurrence and development of RA, affects synovial inflammation^37^, synovial angiogenesis^38^, chondrocyte proliferation, apoptosis and autophagy^39^, and migration and invasion of fibroblast-like synoviocytes in RA^40^. The WB result showed that HDW could inhibit the PI3K/AKT pathway by reducing phosphorylation of AKT in MH7A cells.

Construction of the common target-related PPI network permitted screening of the top 8 core genes: RELA (transcription factor p65), TNF (tumor necrosis factor), IL6 (interleukin-6), TP53 (cellular tumor antigen p53), MAPK1 (mitogen-activated protein kinase 1), AKT1(RAC-α serine/threonine-protein kinase), IL10 (interleukin-10), and ESR1(estrogen receptor). RELA, ranking first with the highest connection in the PPI network, is a subunit of nuclear factor (NF)-κB and is regarded as an important member of the NF-κB pathway^41^. RELA participates in essential life activities, including cell proliferation, transformation, ap-optosis, inflammation and immune response and has been confirmed as an important component of the pathogenesis of RA^42^. TNF is an important therapeutic target for RA and TNF agents were the first molecular targeting drugs developed for the treatment of RA^43,44^. IL6 and IL 10 are critical for the development and progression of RA by affecting the inflammatory process, osteoclast-mediated bone resorption and pannus development^45,46^. RT‐qPCR results showed that RELA, TNF, and IL6 were up‐regulated while IL10 was down‐regulated with HDW treatment. However, there were no significant differences in other four targets, including TP53, MAPK1, AKT1, and ESR1. Studies with TP53, MAPK1, AKT1, and ESR1 have focused on cancer, whereas those on RA have focused on deficiency. Of course, it is possible that the level of mRNA observed may not accurately reflect the level of protein present. As such, whether HDW can directly influence these hub genes requires further study. The antirheumatic effect of compounds such as quercetin and kaempferol is partially associated with these target genes. For example, quercetin, a natural substance, can induce mitochondrial apoptosis of RA-FLSs through the p53 mechanism^47^. These results therefore illustrate the range of interactions between the multi-components and multi-targets of HDW in the treatment of RA.

As noted above, RELA, TNF, IL6, TP53, MAPK1, AKT1, IL10, and ESR1 are the top 8 hub genes in the PPI network. Additionally, however, potential targets were found to be involved in multiple pathways in the KEGG enrichment analysis. This indicates that HDW may exert its anti-RA effects by the combined interaction of multi-pathways and multi-targets. Also, the molecular docking analysis was carried out to investigate the interaction of 5 compounds and 8 targets. For example, the absolute value of binding energy about β-sitosterol and ESR1 is the highest in each group, indicating that β-sitosterol has a higher binding affinity than other target genes. Although there have been relatively few studies with stigmasterol and RA, the docking results indicated that stigmasterol performed good binding activity with TP53, ESR1, and IL10. In brief, the higher the binding affinity of components and targets, the more likely HDW treatment of RA will be achieved by modulating several related targets. However, further experiments are needed to clarify active compounds and mechanisms of action analysis of HDW for RA.

## Methods

### Active components and potential targets of HDW

Active components and potential targets of HDW were selected from the Traditional Chinese Medicine Systems Pharmacology Database and Analysis Platform (TCMSP, https://old.tcmsp-e.com/tcmsp.php)^55^. As recommended by TCMSP, the compounds with OB ≥ 30% have good absorption and slow metabolism after oral administration, while the compounds with DL ≥ 0.18 were chemically suitable for drug development. We therefore used OB ≥ 30% and DL ≥ 0.18 as filtering for thresholds^56^. An exhaustive search for components was performed using PubMed (https://pubmed.ncbi.nlm.nih.gov/) and CNKI (https://www.cnki.net/). The active components of HDW were determined by SwissADME database (http://www.swissadme.ch/). Afterward, the targets (probability ≥ 0.9) corresponding to the components were screened from the SwissTargetPrediction database (http://swisstargetprediction.ch/)^57^. Duplicates were deleted to obtain potential targets for further analysis.

### Potential targets of rheumatoid arthritis

Potential RA-related therapeutic targets were collected using keywords “rheumatoid arthritis” as a query from the OMIM (https://www.omim.org/)^58^, DrugBank (https://go.drugbank.com/)^59^, TTD (http://db.idrblab.net/ttd/)^60^, GeneCards (https://www.genecards.org/)^61^ and DisGeNET (https://www.disgenet.org/) databases^62^. All acquired targets were imported into UniProt (https://www.uniprot.org/) for normalization and removal of duplicate and erroneous targets.

### Common targets and construction of the compound-target-RA network

Targets related to the active components of HDW were mapped to RA-related targets to identify ones common to both. These were considered as targets of HDW on treating RA. Following compilation of these, the “compound-target-RA” network was established using Cytoscape 3.9.0 software to determine the interaction relationships^63^ and to further screen for key active components by calculating the extent of these.

### Gene ontology and pathway enrichment analysis

Gene Ontology (GO) analysis and KEGG pathway analysis were performed using the R 3.6.3 and related R packages (clusterProfiler, Org.Hs.eg.db, and ggplot2)^64^. After adjusting the p-value using the Benjamini–Hochberg (BH) method, P.adjusted values < 0.01 were considered to be statistically significant.

### Network visualization and identification of hub targets

To further identify interactive relationships among the common target proteins, the protein list was mapped to the STRING database (https://cn.string-db.org/)^65^. To ensure reliability, we used a cutoff ≥ 0.9 (high-confidence interaction score) to obtain the significant PPIs in network visualization. The CytoNCA plug-in was used to explore hub targets, and the top 8 were generated using Betweenness (BC), Closeness (CC), Degree (DC), and Network (NC) methods.

### Molecular docking

To investigate the associations between the Active Components and hub targets, we applied molecular docking analysis. The mol2 structure files of the 5 main components were downloaded from TCMSP. The crystal structure of the 8 hub targets were obtained from the Protein Data Bank (PDB, https://www.rcsb.org/). Molecular docking studies were carried out with AutoDock 4.0 software. After docking, the results were carried out by sorting the binding energy predicted by docking conformations. The lower the affinity score is, the better the binding effect with a binding energy of less than − 5.0 kcal/mol defined as dependable binding. Binding modes were visualized with the program Pymol.

### Cell culture and treatment

MH7A, a human RA-FLS cell line, was purchased from Jennio biological technology (Guangzhou, China). The MH7A cell was maintained in DMEM with 10% fetal bovine serum (Gibco, USA), supplemented with 1% penicillin-streptomycin (Hyclone, USA). HDW (Lot. Number: R02S11Y123184) was purchased from Shanghai Yuanye Bio-Technology.

### Cell counting kit-8 (CCK-8) assay

CCK‐8 (Biyuntian, Shanghai, China) assay was used to measure cell proliferation. MH7A cells were seeded into 96-well plates at a cell density of 5 × 10^3^ cells/well and incubated in a 5% CO_2_ incubator at 37 °C. After adhering to the wall, cells were treated with Bavachinin of different concentrations (0, 0.5, 1, and 2 mg/mL) for 48 h. Optical density at 450 nm was measured with a microplate reader after adding the CCK-8 solution and incubated for additional 3 h.

### Quantitative real-time polymerase chain reaction (qRT-PCR)

MH7A cells were seeded in 6-well plates at 5 × 10^6^ cells/well. After 48 h of treatment, cells were collected by trypsinization. Total RNA from MH7A cells and ankle joint tissues were extracted by employing the TRIzol reagent, and cDNA was synthesized using a reverse transcription kit. qPCR was performed using SYBR Green PCR Master Mix (Bio-Rad, USA). Finally, the relative quantity of mRNA was calculated using the 2^−∆∆Ct^ method. The primers (synthesized in Qingke, China) used are listed in the Supplementary Table S4.

### Western blotting (WB)

MH7A cells were cultured in T75 cell culture flask. After 48 h of treatment, cells were lysed with 1 × RIPA lysis buffer containing 1% of PMSF and 1% of phosphotransferase inhibitor. Protein concentration was detected by the BCA Protein Quantitative Kit (Thermo Fisher Scientific, USA). Subsequently, proteins (25 μg) were denatured by employing heating followed by electrophoresis with 10% SDS-PAGE, and were transferred to polyvinylidene fluoride membranes (Millipore, USA). The membranes were sealed with 5% skimmed milk (or 5% BSA) for 2 h at 37 °C, and incubated at 4 °C overnight with the following primary antibodies: protein kinase B (AKT), phospho-Akt (p‐AKT) and β-actin. After incubation, the membrane was further incubated with horseradish peroxidase (HRP)-conjugated secondary antibodies for 1.5 h at 37 °C. Lastly, specific bands were detected using enhanced chemiluminescence reagent (Thermo Fisher Scientific) and calculated by Image J software for the quantitative analysis of proteins.

## Conclusions

In summary, this study explored the therapeutic effect and mechanism of HDW on RA through a strategy combining network pharmacology and in vivo experiment verification. Five active components and 8 core targets of HDW were finally screened. PI3K/AKT signaling pathways were enriched by KEGG analysis in addition to virus and cancer-related pathways. Molecular docking showed that the active ingredients successfully docked with related targets. Through experimental validation, we found that HDW may affect target genes RELA, TNF, IL6, and IL10 through multiple signaling pathways PI3K/AKT signaling pathways, thereby affecting the pathological process of RA and ultimately inhibiting the occurrence and development of RA. Our findings provide a theoretical basis for the use of HDW as a therapeutic for RA.

## Supplementary Information

Supplementary Information.

## Data Availability

All data generated or analysed during this study are included in its supplementary information files.
