# Supplementary material for: Network pharmacology and experimental validation to identify the potential mechanism of Hedyotis diffusa Willd against rheumatoid arthritis
Source: Sci Rep. 2023 Jan 25;13:1425. doi: 10.1038/s41598-022-25579-3 (PMC9877023; doi:10.1038/s41598-022-25579-3)
Supplement: Supplementary file 1 — Supplementary Information. [file 41598_2022_25579_MOESM1_ESM.pdf]

Supplement Table 1. The active components and potential targets of HDW.

PubChem CID	Active Components	Targets
5281330	poriferasterol	PGR, NR3C2
10514946	2-methoxy-3-methyl-9,10-anthraquinone	PTGS1, DRD1, CHRM3, CHRM1, ESR1, SCN5A, CHRM5, PTGS2, CHRM4, RXRA, OPRD1, PDE3A, HRH1, HTR2A, SLC6A2, ADRA1A, HTR2A, CHRM2, ADRA1B, SLC6A3, ADRB2, ADRA1D, SLC6A4, OPRM1, GABRA1, HSP90, PIK3CG, CHRNA7, NCOA2, PKIA
5280794	stigmasterol	PGR, NR3C2, NCOA2, ADH1C, RXRA, NCOA1, PTGS1, PTGS2, ADRA2A, SLC6A2, SLC6A3, ADRB2, PLAU, LTA4H, MOB, MOA, CTRB1, CHRM3, CHRM1, ADRB1, SCN5A, ADRA1A, CHRM2, ADRA1B
222284	β-sitosterol	PGR, NCOA2, PTGS1, PTGS2, KCNH2, CHRM3, CHRM1, SCN5A, CHRM4, PDE3A, ADRA1A, CHRM2, ADRA1B, ADRB2, CHRNA2, SLC6A4, OPRM1, BCL2, BAX, CASP9, CASP3, CASP8, PRKCA, TGFB1, PON1, MAP2
5280343	quercetin	PTGS1, AR, PPARG, PTGS2, HSP90, NCOA2, TOP2, KCNH2, SCN5A, ADRB2, MMP3, F7, RXRA, ACHE, RELA, EGFR, AKT1, VEGFA, CCND1, BCL2, BCL2L1, CDKN1A, BAX, CASP9, PLAU, MMP2, MMP9, MAPK1, IL10, EGF, RB1, TNF, IL6, AHSA1, CASP3, TP53, ELK1, NFKBIA, POR, ODC1, XDH, CASP8, TOP1, RAF1, PRKCA, MMP1, HIF1A, STAT1, RUNX1T1, ERBB2, PPARG, ACACA, HMOX1, CYP3A4, CAV1, MYC, F3，GJA1, CYP1A1, ICAM1, IL1B, CCL2, SELE, VCAM1, PTGER3, PRKCB, BIRC5, DUOX2, NOS3, HSPB1, IL2, NR1I2, CYP1B1, CCNB1, PLAT, THBD, SERPINE1, IFNG, IL1A, MPO, TOP2A, NCF1, HAS2, GSTP1, NFE2L2, AHR, PSMD3, SLC2A4, CXCL11, CXCL2, DCAF5, NR1I3, CHEK2, INSR, CLDN4, PPARA, PPARD, HSF1, CRP, CXCL10, CHUK, SPP1, RUNX2, RASSF1, E2F1, E2F2, ACP3, CTSD, IGFBP3, IGF2, CD40LG, IRF1, ERBB3, PON1,DIO1, PCOLCE, NPEPPS, HK2, NKX3-1, RASA1, GSTM1, GSTM2
5280863	kaempferol	NOX4, AKR1B1, XDH, TYR, FLT3, CA2, ALOX5, CA7, HSD17B2, ABCC1, HSD17B1, AHR, CA12, ESRRA, ABCB1, CYP1B1, ABCG2
5280460	scopoletin	CA7, CA12, CA9

637542	p-Coumaric acid	AKR1B1, CA1, CA2, CA3, CA4, CA9, CA5A, CA5B, CA6, CA7, CA12, CA14, ESR2
72	3，4-Dihydroxybenzoic acid	CA2, CA7, CA1, CA6, CA12, CA14, CA9, CA4
445858	Ferulic acid	CA2, CA7, CA1, CA6, CA12, CA14, CA9, CA5A,
135	p-Hydroxybenzoic acid	CA2, CA7, CA1, CA3, CA6, CA12, CA14, CA9, CA4, CA5B, CA5A, CA13

Supplement Table Table 2. The list of genes contributing to the the 20 selected pathways.

Description	geneID	Count
AGE-RAGE signaling pathway in diabetic complications	AKT1/CCND1/BCL2/CASP3/F3/ICAM1/IL1A/IL1B/IL6/MMP2/NOS3/SERPINE1/MAPK1/RELA/CCL2/SELE/STAT1/TGFB1/THBD/TNF/VCAM1/VEGFA	22
TNF signaling pathway	AKT1/CASP3/CASP8/CHUK/CXCL2/ICAM1/IL1B/IL6/CXCL10/IRF1/MMP3/MMP9/NFKBIA/MAPK1/PTGS2/RELA/CCL2/SELE/TNF/VCAM1	20
Kaposi sarcoma-associated herpesvirus infection	AKT1/CCND1/CASP3/CASP8/CHUK/CXCL2/HIF1A/ICAM1/IL6/MYC/NFKBIA/PIK3CG/MAPK1/PTGS2/RELA/STAT1/TP53/VEGFA	18
IL-17 signaling pathway	CASP3/CASP8/CHUK/CXCL2/IFNG/IL1B/IL6/CXCL10/MMP1/MMP3/MMP9/NFKBIA/MAPK1/PTGS2/RELA/CCL2/TNF	17
Hepatitis C	AKT1/CCND1/CASP3/CASP8/CHUK/EGF/IFNG/CXCL10/MYC/NFKBIA/PPARA/MAPK1/RELA/RXRA/STAT1/TNF/TP53	17
Toxoplasmosis	AKT1/ALOX5/BCL2/CASP3/CASP8/CD40LG/CHUK/IFNG/IL10/NFKBIA/PIK3CG/MAPK1/RELA/STAT1/TGFB1/TNF	16
Hepatitis B	AKT1/BIRC5/BCL2/CASP3/CASP8/CHUK/IL6/MMP9/MYC/NFKBIA/MAPK1/RELA/STAT1/TGFB1/TNF/TP53	16
Influenza A	AKT1/CASP3/CASP8/CHUK/ICAM1/IFNG/IL1A/IL1B/IL6/CXCL10/NFKBIA/MAPK1/RELA/CCL2/STAT1/TNF	16
Human cytomegalovirus infection	AKT1/CCND1/CASP3/CASP8/CHUK/IL1B/IL6/MYC/NFKBIA/MAPK1/PTGS2/RELA/CCL2/TNF/TP53/VEGFA	16
PI3K-Akt signaling pathway	AKT1/CCND1/BCL2/CHUK/EGF/IL2/IL6/MYC/NOS3/PIK3CG/MAPK1/RELA/RXRA/SPP1/TP53/VEGFA	16
Tuberculosis	AKT1/BCL2/CASP3/CASP8/CTSD/IFNG/IL1A/IL1B/IL6/IL10/MAPK1/RELA/STAT1/TGFB1/TNF	15
Epstein-Barr virus infection	AKT1/CCND1/BCL2/CASP3/CASP8/CHUK/ICAM1/IL6/CXCL10/MYC/NFKBIA/RELA/STAT1/TNF/TP53	15
Proteoglycans in cancer	AKT1/CCND1/CASP3/CAV1/ESR1/HIF1A/MMP2/MMP9/MYC/PLAU/MAPK1/TGFB1/TNF/TP53/VEGFA	15

Human papillomavirus infe ction	AKT1/CCND1/CASP3/CASP8/CHUK/EGF/IRF1/MAPK1/PTGS2/RELA/SPP1/STAT1/TNF/TP53/VEGFA	15
Prostate cancer	AKT1/AR/CCND1/BCL2/CHUK/EGF/GSTP1/MMP3/MMP9/NFKBIA/PLAU/MAPK1/RELA/TP53	14
C-type lectin receptor signal ing pathway	AKT1/CASP8/CHUK/IL1B/IL2/IL6/IL10/IRF1/NFKBIA/MAPK1/PTGS2/RELA/STAT1/TNF	14
MicroRNAs in cancer	CCND1/BCL2/CASP3/CYP1B1/HMOX1/MMP9/ABCC1/MYC/ABCB1/PLAU/MAPK1/PTGS2/TP53/VEGFA	14
Herpes simplex virus 1 infe ction	AKT1/BCL2/CASP3/CASP8/CHUK/IFNG/IL1B/IL6/NFKBIA/RELA/CCL2/STAT1/TNF/TP53	14
Toll-like receptor signaling pathway	AKT1/CASP8/CHUK/IL1B/IL6/CXCL10/NFKBIA/MAPK1/RELA/CXCL11/SPP1/STAT1/TNF	13
Th17 cell differentiation	AHR/CHUK/HIF1A/IFNG/IL1B/IL2/IL6/NFKBIA/MAPK1/RELA/RXRA/STAT1/TGFB1	13

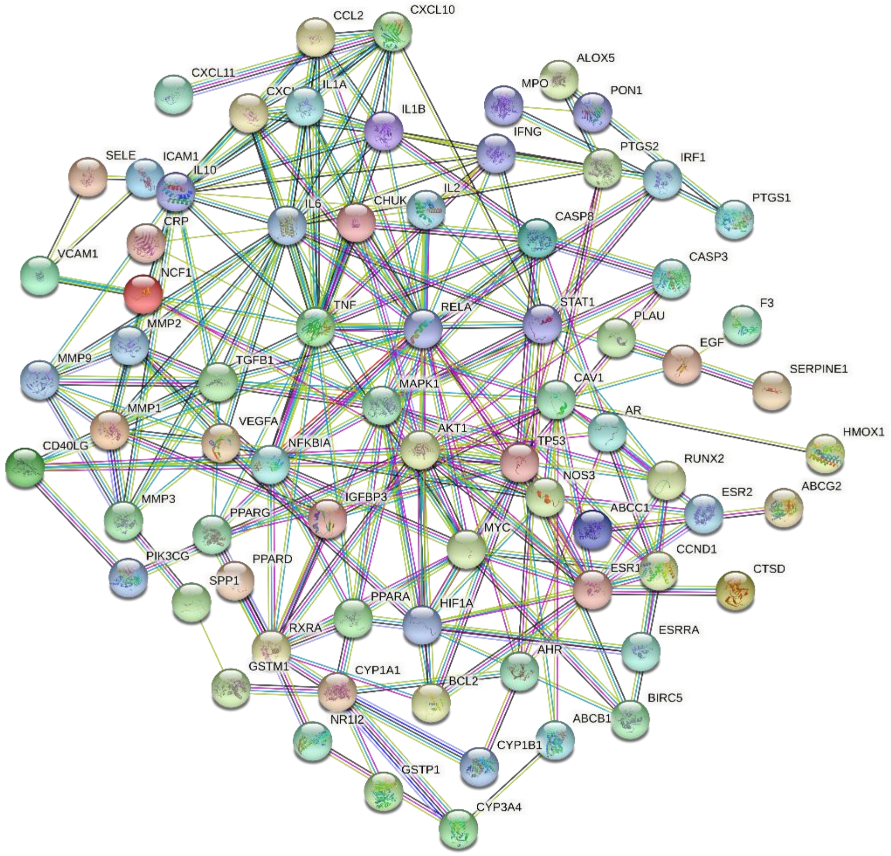

Supplement Figure 1. The PPI network of 85 common targets.

Supplement Table 3. Hub targets of HDW against RA.

Uniprot ID	Gene Symbol	Description
Q04206	RELA	Transcription factor p65
P01375	TNF	Tumor necrosis factor
P05231	IL6	Interleukin-6
P04637	TP53	Cellular tumor antigen p53
P28482	MAPK1	Mitogen-activated protein kinase 1
P31749	AKT1	RAC-alpha serine/threonine-protein kinase
P22301	IL10	Interleukin-10
P03372	ESR1	Estrogen receptor

Supplement Table 4. Primer sequences for all genes.

Gene Symbol	Gene Symbol	Sequences (5′-3′)
RELA	F	CTGTCCTTTCTCATCCCATCTT
	R	TCCTCTTTCTGCACCTTGTC
TNF	F	CCAGGGACCTCTCTCTAATCA
	R	TCAGCTTGAGGGTTTGCTAC
IL6	F	ACAGCCACTCACCTCTTCAG
	R	GCAAGTCTCCTCATTGAATCCA
TP53	F	GTACCACCATCCACTACAACTAC
	R	CACAAACACGCACCTCAAAG
MAPK1	F	GGTACAGGGCTCCAGAAATTAT
	R	TGGAAAGATGGGCCTGTTAG
AKT1	F	CTTCTATGGCGCTGAGATTGT
	R	GCCCGAAGTCTGTGATCTTAAT
IL10	F	CTAACCTCATTCCCCAAC
	R	GACCTCAAGTGATCCACC
ESR1	F	AGGGTGGCAGAGAAAGATTG
	R	GGTAGCCTGAAGCATAGTCATT
β-actin	F	ACTTAGTTGCGTTACACCCTT
	R	GTCACCTTCACCGTTCCA

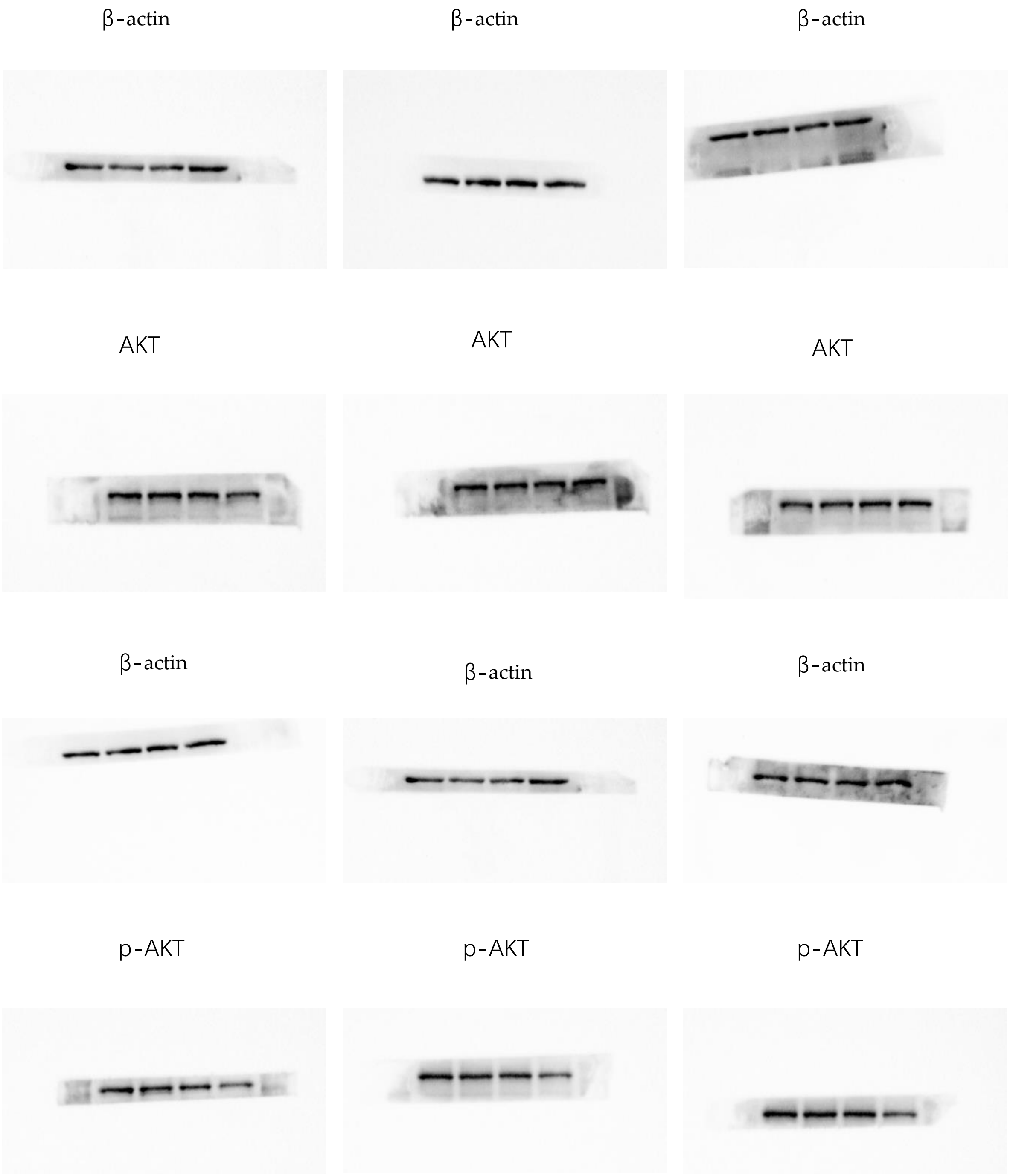

Supplement Figure 2. The original immunoblot images of AKT, p-AKT, and β-actin. The experiment was independently repeated three times.
